# Access site complications following Impella-supported high-risk percutaneous coronary interventions

**DOI:** 10.1038/s41598-019-54277-w

**Published:** 2019-11-28

**Authors:** Laura Johannsen, Amir A. Mahabadi, Matthias Totzeck, Andrea Krueger, Rolf Alexander Jánosi, Tienush Rassaf, Fadi Al-Rashid

**Affiliations:** Department of Cardiology and Vascular Medicine, West German Heart and Vascular Center Essen, University Hospital Essen, Medical Faculty, University, Duisburg-Essen, Germany

**Keywords:** Interventional cardiology, Cardiac device therapy

## Abstract

Mechanical circulatory support (MCS) devices are increasingly used to provide hemodynamic stability for patients with severe coronary artery disease, comorbidities, and/or impaired hemodynamics during high-risk percutaneous coronary interventions (PCI). Vascular access site complications, particularly those due to the use of large-bore sheaths, may limit outcomes in these patients. The aim of this study was to investigate the incidence and predictors of vascular complications in protected high-risk PCIs. Therefore, we included patients undergoing high-risk PCI with an Impella device from January 2016 to August 2018. Vascular complications were graded according to ‘Valve Academic Research Consortium-2’, a definition routinely used in transcatheter valve implantation procedures. In total, 61 patients (mean age 72 ± 11 years, 79% male, SYNTAX score 33 ± 7) were included, and angiographic- and fluoroscopic-guided vascular access was used for Impella implantation in all patients. Major vascular complications occurred in 5 male patients (8%). All major vascular complications were treated conservatively without the need for surgical intervention, and only one patient received a transfusion of three erythrocyte concentrates. Regression analysis revealed that patients with peripheral arterial disease of the lower extremities are at higher risk of major vascular complications. In conclusion, the utilization of Impella using a standardized protocol for angiographic- and fluoroscopic-guided vascular access was associated with a low rate of vascular complications.

## Introduction

Mechanical circulatory support (MCS) devices provide hemodynamic support during high-risk percutaneous coronary interventions (PCIs)^1,2^. Among all currently available MCS devices, the Impella left-ventricular assist device is predominantly used^3–6^. In addition to its hemodynamic benefits, using vascular access to the femoral artery with large bore-sheaths increases the risk of vascular complications, including bleeding, which is an established predictor of mortality^7,8^. Registry data and single-center studies have reported varying rates of vascular complications from 3.4–33% when MCS devices are used^6,9,10^. When the Impella device was compared to the Intra-Aortic Balloon Pump, a more than two-fold higher rate of bleeding complications was observed in patients with cardiogenic shock^11^. Most studies do not use a standardized definition for vascular complications and predominantly focus on adverse vascular events that require the transfusion of erythrocyte concentrates or surgical treatment^4,6,12^. Moreover, complications, such as hematomas, are often not reported^5,9^. The aim of this study was therefore to assess the incidence and predictors of vascular complications using a standardized definition for vascular complications as defined by the Valve Academic Research Consortium-2 (VARC-2)^13^ in patients undergoing high-risk PCI with Impella support.

## Methods

### Study design and population

We included consecutive patients who underwent PCI with MCS from January 2016 to August 2018 at our tertiary care center. The decision to use an Impella implantation device was based on a “Heart Team-based” algorithm that used the best available evidence to reach an individualized treatment decision. Details about the cohort were previously published^14^. This algorithm incorporated the anatomical lesion complexity (defined by the SYNTAX I score), comorbidities (oxygen-dependent chronic obstructive pulmonary disease, severe aortic valve stenosis III°, carotid artery disease, chronic kidney disease stage ≥4, severe pulmonary hypertension, peripheral artery disease stage 4, stroke within 30 days prior to PCI, active infection/sepsis and cancer with concurrent cancer therapy), and clinical presentation, including hemodynamic status (left-ventricular ejection fraction), to identify patients at high-risk of coronary interventions.

**Figure 1:**
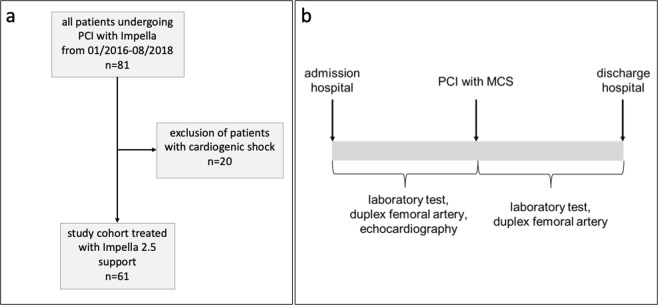
Flow-chart of the study (**a**) with an overview of the timeline and the different examinations performed. (**b**) MCS: mechanical circulatory support; PCI: percutaneous coronary intervention.

We focused on patients who underwent Impella-supported high-risk PCI in the present study (Fig. 1). Patients with cardiogenic shock and those presenting with ongoing cardiopulmonary resuscitation (prior to coronary angiography) were excluded from the study. All patients received an Impella device (Abiomed, Danvers, MA, USA) for MCS. The study was approved by the institutional ethics committee of the University of Duisburg-Essen (Essen, Germany - 18-8337-BO). All procedures were performed in accordance with relevant guidelines and regulations^2,15,16^. All patients gave written informed consent for study participation and publication, and the study conformed to the principles of the Declaration of Helsinki.

### Procedural characteristics

Data on the laboratory values, risk factors and clinical diagnoses of patients were obtained from all available hospital records. All patients received pre- and postinterventional angiological examinations, including ultra- and doppler-sonography of the lower extremity arteries and preinterventional transthoracic echocardiography (Fig. 1). Ultrasonography of the lower extremity arteries included assessments of stenosis, hematoma and pseudoaneurysm.

Vascular access for MCS was conducted by fluoroscopic- and angiographic-guided femoral artery puncture proximal to the femoral artery bifurcation. Vascular closure was performed with vascular closure devices using the preclosure technique with a Proglide system (Abbott Laboratories, Redwood City, USA)^17^. Prior to Impella sheath placement, peripheral angiography (through the contralateral femoral access selected for PCI) was performed to identify the optimal target area proximal to the femoral artery bifurcation. Accordingly, when the atherosclerotic burden or tortuosity was high, the contralateral iliac-femoral axis was assessed, and the most favorable side was chosen for Impella implantation. After the needle was introduced into the tissue tract, fluoroscopy was performed to ensure that the needle tip was within the optimal target area, and arterial entry was performed via the insertion of a 6-French sheath. A single Proglide device was deployed with readvancement of the wire via the monorail wire port on the shaft. Subsequently, an 8-French sheath was inserted, and the preloaded sutures were safeguarded on the drapes with a hemostat. The 8-French sheath was then exchanged for a large-bore Impella introducer sheath using a stiff guidewire (Amplatz-Superstiff, Boston Scientific, Massachusetts, USA). After sheath insertion, heparin was administered to achieve an activated clotting time > 250 s, which was maintained throughout the entire procedure with intravenous heparin therapy^18^. At the end of the procedure, the Impella introducer sheath was fully removed under manual compression, leaving the guidewire in the vessel. The preformed Proglide knot is advanced to the arteriotomy site using a knot pusher, and the wire was removed if hemostasis was reached. Additionally, a pressure bandage was applied in all patients for the following 6 hours.

### Study outcome measures

The primary study endpoint was the incidence of vascular complications. For this analysis, we specified major and minor vascular complications according to VARC-2^13^. The secondary endpoint was the identification of possible predictors of major vascular complications.

### Variable definitions

Major vascular complications were defined as access site- or access-related vascular injury leading to death, life-threatening or major bleeding (a drop in the hemoglobin level of at least 3.0 g/dl or requiring transfusion of two or three erythrocyte concentrates), visceral ischemia or the use of unplanned endovascular or surgical intervention. Access site- or access-related vascular injury that did not meet the criteria for major vascular complications were granted as minor vascular complication^13^. Data on vascular complications were obtained from all available hospital records, laboratory values and duplex sonographic findings obtained in the lower extremities. The rates of vascular complications were reported after stratification by VARC-2 criteria into minor and major vascular complications.

Peripheral artery disease was defined according to European guidelines^19^. Patients with peripheral artery disease (of the lower extremities) at Fontaine stage IV (including patients with ulceration and gangrene) did not receive an Impella device for hemodynamic support.

Dual anti-platelet therapy was defined as therapy with acetylsalicylic acid and clopidogrel or ticagrelor. Triple therapy was defined as simultaneous therapy with acetylsalicylic acid, (new) oral anticoagulant, and clopidogrel.

### Historical cohort

The historical control patients were identified from 2 studies: the Europella Registry^9^ and a historical cohort from our tertiary center^20^. The study cohort was compared to the Europella registry (n = 144) with respect for serious adverse vascular events that required the transfusion of erythrocyte concentrates or interventional/surgical treatment. A comparison regrading according to VARC-2 criteria was not possible due to missing laboratory values.

The vascular complications in the historical Essen cohort (n = 38) were regraded according to the VARC-2 criteria, and major vascular complications were compared.

### Statistical analysis

Data are presented as the mean ± standard deviation if normally distributed or as medians and interquartile ranges otherwise. Categorical variables are presented as frequencies and percentages. Categorical data were compared between groups using the χ^2^ test. Continuous variables were compared using Student’s t-test if normally distributed or the Mann–Whitney U test if not. Logistic regression analysis was used to evaluate the association of patients‘ and procedural characteristics with major vascular complications. Effect sizes are depicted as odds ratios (ORs) and 95% confidence intervals (CIs). For continuous variables, effect sizes were calculated per standard deviation change. A p-value of < 0.05 indicated statistical significance. All analyses were performed using SPSS software (version 25, SPSSS, Chicago, IL, USA).

## Results

### Baseline and procedural characteristics

We included a total of 61 consecutive patients (mean age 72 ± 11 years, 79% male, SYNTAX score 33 ± 7) who underwent high-risk PCI with Impella 2.5. Twenty-four patients (39%) presented with acute coronary syndrome. Nearly half of all patients (n = 30, 49%) had antiplatelet therapy with aspirin before beginning the PCI. The majority of the patients (n = 53, 87%) underwent multivessel PCI. Baseline and procedural characteristics are given in Table 1. All patients were weaned from MCS devices in the catheterization laboratory, and vascular closure was performed after removal of the Impella Peel-away sheath (12 F) with one Proglide using the preclosure technique in all of these cases.

**Table 1:** Baseline and peri- and postprocedural characteristics.

	All patients (n = 61)
**Baseline characteristics**	
male sex, n (%)	48 (79)
age [yrs.]	72 ± 11
body mass index [kg/m²]	28 ± 5
hypertension, n (%)	50 (82)
acute coronary syndrome, n (%)	24 (39)
ST elevation myocardial infarction, n (%)	3 (5)
non-ST elevation myocardial infarction, n (%)	13 (21)
unstable angina pectoris, n (%)	8 (13)
baseline hemoglobin [mg/dl]	13 ± 2
anemia, n (%)	23 (38)
diabetes mellitus, n (%)	23 (38)
CAD with prior revascularization, n (%)	29 (48)
prior coronary bypass surgery, n (%)	7 (12)
LV-EF [%]	43 ± 12
peripheral artery disease, n (%)	13 (21)
(new) oral anticoagulant, n (%)	9 (15)
prior vascular complications, n (%)	0
common femoral artery diameter [mm]	8 ± 1
logistic EuroSCORE [%]	11 ± 14
SYNTAX score [%]	33 ± 7
**Periprocedural characteristics**	
PCI left main coronary artery, n (%)	48 (79)
PCI left anterior descending artery, n (%)	53 (87)
PCI left circumflex coronary artery, n (%)	46 (75)
PCI right coronary artery, n (%)	9 (15)
PCI bypass graft, n (%)	2 (3)
multivessel PCI	53 (87)
contrast agent [ml]	271 ± 107
GP IIa/IIb inhibitor	2 (3)
**Postprocedural characteristics**	
acetylsalicylic acid and ticagrelor	27 (44)
acetylsalicylic acid and clopidogrel	24 (39)
triple anticoagulant therapy	10 (16)

All continuous variables are presented as the mean** ± **standard deviation. CAD: coronary artery disease; LV-EF: left ventricular ejection fraction; PCI: percutaneous coronary intervention; triple therapy: (new) oral anticoagulant, acetylsalicylic acid and clopidogrel.

### Access site complications

The rate of major vascular complications, including major bleeding from the puncture site, was low (n = 5, 8%). All major vascular complications were treated conservatively without the need for surgical intervention, and only one patient received a transfusion of three erythrocyte concentrates. Table 2 shows the detailed characteristics of all major vascular complications. In addition to major vascular complications, minor vascular complications were observed in 22 patients (36%), with superficial hematoma reported in 95% of the cases. There was no need for any further intervention or treatment in the patients with minor vascular complications.

**Table 2:** Detailed description of access site-related major vascular complications according to the Valve Academic Research Consortium-2.

Patient number	Event description	Baseline Hb [g/dL]	∆Hb [g/dL]	Treatment
20	inguinal hematoma, decrease in hemoglobin ≥3 g/dl	12.5	3	conservative
27	bleeding from puncture site	14.6	3	conservative
40	inguinal hematoma, decrease in hemoglobin ≥3 g/dl	13.6	3.5	conservative
41	bleeding from puncture site, decrease in hemoglobin ≥3 g/dl	11.1	5.6	transfusion of three erythrocyte concentrates
85	bleeding from puncture site, decrease in hemoglobin ≥3 g/dl	13.4	4.1	conservative

Hb: hemoglobin; ∆Hb was measured by the initial value and the lowest recorded Hb- value until 72 h post-procedure.

Emergency patients had a non-significantly higher rate of major vascular complications (emergent n = 3; 12.5% vs. scheduled n = 2; 5.4%; p = 0.33). Patients with preprocedural anti-platelet therapy (major vascular complications: 10% vs. no vascular complication: 6.5%; p = 0.62) or oral anticoagulation (major vascular complications 10% vs. no vascular complication 7.8%; p = 0.82) showed no significant difference in the occurrence of vascular complications. In addition, there was no significant difference in the diameter of the common femoral artery between patients with and without major vascular complications (7.9 ± 0.9 mm vs. 8.4 ± 1.5 mm; p = 0.5). In a regression analysis (Table 3), peripheral arterial disease was associated with a higher rate of major vascular complications (OR: 6.90, CI 95% 1.02; 48.65, p = 0.048).

**Table 3:** Regression analysis to determine risk factors for major vascular complications in patients treated with mechanical circulatory support.

	Univariate regression analysis		Multivariate regression analysis	
	OR (CI 95%)	p-value	OR (CI 95%)	p-value
age	0.95 (0.87;1.03)	0.21		
anemia	0.39 (0.04;3.69)	0.41		
female sex*				
peripheral arterial disease	6.90 (1.02, 48.65)	0.04		
triple anticoagulant therapy	1.15 (0.12; 11.42)	0.91		

*No event (major vascular complication).

### Comparison to historical control

The rate of major vascular complications according to the VARC-2 criteria was significantly lower in our cohort (8% [n = 5/61]) than in the historical Essen cohort (34% [n = 13/38], p = 0.001). With regard for serious adverse vascular events requiring the transfusion of erythrocyte concentrates or interventional/surgical treatment, the Europella study observed a higher rate than was found our study [10.4% (n = 15/144) vs. 1.6% (n = 1/61); p = 0.03]. The incidence of requirement of blood transfusion was low in our cohort (1.6%; n = 1/61). In comparison to the Europella Registry (5.6%; n = 8/144), in our cohort, there was a nonsignificant (p = 0.163) trend towards a lower rate.

## Discussion

The findings of our study are as follows: 1. the rate of major vascular complications according to VARC-2 definitions was low in patients treated with Impella devices, 2. all vascular complications can be treated conservatively without the need for interventional or surgical treatment, and 3. peripheral arterial disease was associated with a higher rate of major vascular complications in our cohort.

Currently, clinical trials investigating the use of MCS describe vascular complications based on varying definitions, most of which reported only severe adverse events^3–6,9,10^. There are currently limited data on minor complications, such as access site hematomas^5,9^. For the systematic analysis of vascular access site complications, we used a standardized definition commonly applied in transcatheter aortic valve implantation procedures - the VARC-2 definition^13^. The key advantage of this definition is that both the need for transfusion and a decrease in hemoglobin levels are included. This enables the occurrence of vascular complications, such as bleeding, in patients with high baseline hemoglobin levels who do not require transfusion despite significant hemoglobin loss.

The rate of major vascular complications was 8% (n = 5) in our cohort according to the VARC-2 definition. Without the use of this definition, 4 of 5 (80%) major vascular complications would not have been defined as major. All patients could be treated conservatively with a watchful waiting strategy except for one patient who received a transfusion of erythrocyte concentrates. Even if these patients could be treated without the need for surgical intervention, these complications led to a prolonged hospital stay. Both the Europella registry (n = 144)^9^ and the USpella registry (n = 175)^6^ investigated the safety and feasibility of elective Impella utilization in high-risk PCIs. In both studies, only complications requiring the transfusion of erythrocyte concentrates (USpella 3.4%, Europella: 6%) and vascular complications such as pseudoaneurysm, fistula and vessel dissection (Europella: 4%, USpella: 3.4%) were reported^6,9^. Regarding the patients’ age, sex and comorbidities, such as diabetes and peripheral arterial disease, our cohort was comparable to that of the Europella registry^9^. With regard for adverse vascular events requiring the transfusion of erythrocyte concentrates or interventional/surgical treatment, the Europella study observed a higher rate [10.4% (n = 15/144) vs. 1.6% (n = 1/61)] of such serious vascular complications than was found in our study. The incidence of requirement of blood transfusion was low in our cohort (1.6%; n = 1/61). In our study, only a single patient required a transfusion due to a drop in hemoglobin of more than 3 g/dl. In comparison to the Europella Registry (5.6%; n = 8/144), there was no significant difference. It is worth noting that the modes of vascular access and closure were not described in the Europella Registry (due to the multicenter and registry design).

In our study, we used fluoroscopic- and angiographic-guided femoral access, which is known to reduce femoral complications^21^. We compared our study cohort to that included in a historical Essen cohort that included patients undergoing high-risk PCI with Impella support^20^. In this cohort, Impella was placed without a standardized protocol for angiographic- and fluoroscopic-guided vascular access. The Impella device was also removed in the catherization laboratory but without the use of a vascular closure device, such as a Proglide. The rate of major vascular complications according to the VARC-2 criteria was significantly lower in our cohort (8% [n = 5/61]) than in the historical Essen cohort (34% [n = 13/38], p = 0.001). The low rate of major vascular complications observed in our study may therefore be attributable to the standardized approach for vascular access.

Several factors can lead to an increased femoral bleeding risk in patients undergoing PCI; these include sheath size, increasing age, a female sex and GPIIb/IIIa inhibitors^22–24^. Interestingly, in our cohort, major vascular complications occurred only in male patients. GPIIb/IIIIa inhibitors were only used in two patients in whom no vascular complications were detected. Our analysis demonstrated an increased risk of major vascular complications associated with peripheral arterial disease (OR: 6.90; CI 95% 1.02, 48.65). These results are in accordance with those of a recent meta-analysis, which reported that the risk of bleeding complications was two-fold higher in patients with peripheral arterial disease than in those without peripheral arterial disease among patients with non-ST elevation acute coronary syndromes^24^. However, the odds ratio was certainly overestimated due to the low number of events (major vascular complications).

### Limitations

The present study has several limitations. The analysis is based on a single-center cohort of a limited number of patients, and data were compared to those in historical cohorts. Therefore, these data should be considered hypothesis-generating. Operator experience may be a potential confounder. Our observational data could only be used to report associations. Causal relations could not be inferred.

## Conclusion

The use of an Impella device during high-risk PCI was associated with a low rate of major vascular complications. Careful patient selection with comprehensive analysis of the femoral access procedure could assist in reducing the incidence of clinically relevant vascular complications.
